# The Association of Depression and Anxiety with Pain: A Study from NESDA

**DOI:** 10.1371/journal.pone.0106907

**Published:** 2014-10-15

**Authors:** Eric W. de Heer, Marloes M. J. G. Gerrits, Aartjan T. F. Beekman, Jack Dekker, Harm W. J. van Marwijk, Margot W. M. de Waal, Philip Spinhoven, Brenda W. J. H. Penninx, Christina M. van der Feltz-Cornelis

**Affiliations:** 1 TopClinical Center for Body, Mind, and Health, GGz Breburg Tilburg, Tilburg, The Netherlands; 2 Tilburg School of Behavioral and Social Sciences, Tranzo Department, University of Tilburg, Tilburg, The Netherlands; 3 Department of Psychiatry, EMGO Institute for Health and Care research, VU University Medical Centre, Amsterdam, The Netherlands; 4 GGZ inGeest, Mental Health Institute, Amsterdam, The Netherlands; 5 Arkin, Mental Health Institute, Amsterdam, The Netherlands; 6 Department of Clinical Psychology, VU University, Amsterdam, The Netherlands; 7 Department of General Practice, EMGO Institute for Health and Care Research, VU University Medical Centre, Amsterdam, The Netherlands; 8 Department of Public Health and Primary Care, Leiden university Medical Centre, Leiden, The Netherlands; 9 Institute of Psychology, Leiden University, Leiden, The Netherlands; 10 Department of Psychiatry, Leiden University Medical Centre, Leiden, The Netherlands; 11 Trimbos Institute, Utrecht, the Netherlands; National Cheng Kung University Medical College, Taiwan

## Abstract

Chronic pain is commonly co-morbid with a depressive or anxiety disorder. Objective of this study is to examine the influence of depression, along with anxiety, on pain-related disability, pain intensity, and pain location in a large sample of adults with and without a depressive and/or anxiety disorder. The study population consisted of 2981 participants with a depressive, anxiety, co-morbid depressive and anxiety disorder, remitted disorder or no current disorder (controls). Severity of depressive and anxiety symptoms was also assessed. In separate multinomial regression analyses, the association of presence of depressive or anxiety disorders and symptom severity with the Chronic Pain Grade and location of pain was explored. Presence of a depressive (OR = 6.67; P<.001), anxiety (OR = 4.84; P<.001), or co-morbid depressive and anxiety disorder (OR = 30.26; P<.001) was associated with the Chronic Pain Grade. Moreover, symptom severity was associated with more disabling and severely limiting pain. Also, a remitted depressive or anxiety disorder showed more disabling and severely limiting pain (OR = 3.53; P<.001) as compared to controls. A current anxiety disorder (OR = 2.96; p<.001) and a co-morbid depressive and anxiety disorder (OR = 5.15; P<.001) were more strongly associated with cardio-respiratory pain, than gastro-intestinal or musculoskeletal pain. These findings remain after adjustment for chronic cardio respiratory illness. Patients with a current and remitted depressive and/or anxiety disorder and those with more severe symptoms have more disabling pain and pain of cardio-respiratory nature, than persons without a depressive or anxiety disorder. This warrants further research.

## Introduction

Chronic pain is common in up to 70% of patients with depressive and anxiety disorders [1]–[9]. Chronic pain and depression most likely have a bidirectional association: depression is a predictor of persistent pain and pain is a predictor of the persistence of depression [1], [3], [10]. A possible explanation is that impaired functioning caused by pain can lead to social isolation, which in turn can lead to a negative effect on depressive symptoms, and vice versa [11], [12]. Furthermore, different brain areas, such as the amygdala and hypothalamus, play a role in both depression and pain [13], [14]. Also, when depression and chronic pain are co-morbid, recognition and treatment of depression are less effective, as patients mostly only present their physical complaints and receive treatment accordingly [1].

Most studies up to now have only considered the relationship of pain with depression, whereas its association with anxiety disorders has been less examined. It is likely that the association of pain and anxiety is equally important, as depression and anxiety commonly appear together. Pain may cause feelings of anxiety, which in turn can make one more sensitive to pain, with persistence of the pain experience as a consequence [15]. Furthermore, anxiety disorders and chronic pain share underlying cognitive and behavioural processes, such as increased attention towards threat and anxious avoidance of physical exertion [16], [17]. Fear avoidance can play a role in chronic pain, with the (acute) pain experience leading to pain catastrophising and pain-related fear which in turn will lead to greater disability and persistent pain experience [15]. Therefore, we need more comprehensive insight by studying both depression and anxiety in concert (separately and as co-morbid problems) with pain [18]–[20]. Another reason to study the cross-sectional relationship between depressive and anxiety disorders and pain is that pain also has a negative impact on the prognosis of psychopathology and psychiatric treatment outcome, with pain leading to more treatment resistance [2], [21]–[23]. Pain may be a marker of a more difficult-to-treat disorder, and lead to a longer time before remission [24].

Pain is a common presenting symptom in depression and anxiety and several studies have explored this association for specific pain symptoms, such as back pain [25]–[27] or neck pain [27], [28]. However, pain symptoms often occur in more than one location and thus may be clustered; clustering of (medically unexplained) physical symptoms was examined by Wessely et al. [29], Nimnuan et al. [30], and Fink et al. [31]. These studies found different clusters of pain symptoms, the most prominent being musculoskeletal, gastro-intestinal, and cardio-respiratory pain. Associations were found between depressive, but mostly anxiety symptoms and cardio-respiratory pain, musculoskeletal pain and gastro-intestinal pain. However, the strength of these associations and the correlation with pain-related disability has not yet been explored [32]–[34]. Therefore, this study will explore the association of clustered locations of pain (musculoskeletal, gastro-intestinal, and cardio-respiratory) with depression and anxiety, while taking severity of pain and pain-related disability into account.

We aim to examine and compare the impact of current and remitted depressive, anxiety and co-morbid disorders on different pain-variables in a large sample of individuals with depressive and/or anxiety disorders versus normal controls. We will explore if severity of depressive or anxiety symptoms is associated with severity of pain and pain-related disability, and whether these associations are stronger for certain clustered pain locations. We expect that not only a depressive disorder will have a strong association with abovementioned pain-variables, but that an anxiety disorder will show a comparably strong association, with comorbid depression and anxiety showing the strongest association.

## Methods

### Sample

The present study used data from the Netherlands Study of Depression and Anxiety (NESDA): an ongoing longitudinal cohort study in which 2981 participants, recruited from the community, general practice and secondary mental health care, are monitored to investigate the long-term course and consequences of depressive and anxiety disorders. Penninx et al. [35] provide a detailed description of the NESDA study design and sampling procedures. NESDA was designed to include patients with depressive and anxiety disorders at different stages of development of their disorder. In order to achieve this, participants were recruited from the community, in primary care and in specialised mental health care [35]. At baseline, healthy controls, persons with a prior history, and persons with a current depressive and/or anxiety disorder, between 18 and 65 years old, were included. The sample was stratified for setting (community, primary care, and specialised mental health). Furthermore, the sample includes a range of psychopathology: from those without a depressive or anxiety disorder (controls) to those with a current, first or recurrent (in the past 6 months) depressive or anxiety disorder and those with a remitted disorder (at baseline, a depressive and/or anxiety disorder was diagnosed in the past, but no diagnoses were present at 6 months before baseline). The disorders included dysthymia, major depressive disorder, general anxiety disorder, panic disorder, social phobia, and agoraphobia. Exclusion criteria were not being fluent in Dutch and a primary diagnosis of psychotic, obsessive compulsive, bipolar or severe addiction disorder. The research protocol was approved by the Ethical Committee of participating universities and written informed consent was obtained from all participants. Interviews were conducted by specially trained research staff.

The sample consists of 2329 persons with a current diagnosis of depression (n = 396) or anxiety disorder (n = 543), a remitted disorder (n = 628) and 652 persons without a history of depressive and/or anxiety disorder and no current diagnosis of depression or anxiety disorder. All 2981 participants were interviewed, by specially trained clinical research staff, for depression and/or anxiety in the baseline interview using the DSM IV based Composite International Diagnostic Interview (CIDI, version 2.1), a reliable and valid instrument for assessing depressive and anxiety disorders [36].

The baseline measurement of NESDA, collected between September 2004 and February 2007, was used for this study. Next to the structured interview to assess mental health, self-report questionnaires were used to assess physical health (such as chronic disease, pain, and severity of mental health).

### Measures

#### Pain assessment

Pain was assessed using the 7-item Chronic Pain Grade (CPG) Scale by von Korff [37]. The CPG is a reliable and valid instrument for chronic pain populations and the general population [37], [38]. The CPG is a good instrument for measuring pain-related variables and for making a hierarchical classification of pain intensity and pain-related disability. It has good internal consistency (with a Cronbach’s alpha of 0.91) and the item-total correlations are all high, ranging from .69 to .83.

The CPG grades (chronic) pain using pain intensity and pain-related disability. Pain intensity is based on the mean of the average, worst, and present pain on a scale of 0–100. Pain–related disability is based on the mean of interference with usual activities, work/household activities, and family/social activities on a scale of 0–100, and the number of days (0–180) one is unable to carry out usual activities due to pain in the previous 6 months. To create 5 grades for chronic pain, the following calculations were used: pain intensity was divided into low intensity (score <50) and high intensity (score ≥50). To calculate the score of pain-related disability, an overall score of 0–6 was created by assigning 0–3 points for disability score (0–29 = 0; 30–49 = 1; 50–69 = 2; 70–100 = 3) and adding 0–3 points for number of disability days (0–6 days = 0; 7–14 days = 1; 15–30 days = 2; >30 days = 3). With these scores, 5 grades of chronic pain can be calculated:

grade 0 (no pain symptoms);
grade 1 (low pain intensity (<50) – low disability (<3 points));
grade 2 (high intensity (≥50) – low disability (<3 points));
grade 3 (high disability – moderately limiting (3–4 disability points, regardless of intensity));
grade 4 (high disability – severely limiting (5–6 disability points, regardless of intensity)).

Along with the CPG, we also assessed the specific pain location. To locate the specific pain location, an inventory was made, with a self-report questionnaire, of pain symptoms in the back, neck, head, stomach, joints, chest, and face. Participants could report one or more of these pain locations, and were asked which of these pain locations bothered them the most in the last six months. We then categorised these pain locations as musculoskeletal (back, neck, head, joints, face), gastro-intestinal (stomach), and cardio respiratory (chest) pain symptoms. Participants could report multiple pain symptoms across the categories.

#### Depression and anxiety

The presence of a depressive or anxiety disorder was established using the CIDI. In this study, psychopathology profiles were made for each participant. A participant either had no psychopathology (n = 652), a remitted disorder (depression and/or anxiety) (n = 628), a current depressive disorder (n = 396), a current anxiety disorder (n = 543) or a current co-morbid depressive and anxiety disorder (n = 762) (in the past 6 months).

In addition to this categorical approach of disorders (yes/no), we also assessed severity of depressive and anxiety symptoms. Severity of depressive symptoms was assessed with the Quick Inventory of Depressive Symptomatology-Self-Report (QIDS-SR) [39], in which no pain items are included. The QIDS-SR is the shortened version of the self-rated Inventory of Depressive Symptomatology (IDS-SR) [40] and is a 16-item questionnaire with a range of 0 to 27 with high internal consistency (Cronbach’s α  = 0.86). A score of 0–5 refers to none to mild depressive symptoms, a score of 6–10 refers to mild severity, a score of 11–15 refers to moderate severity, and a score of 16 or higher refers to (very) severe depressive symptoms. Severity of anxiety symptoms was assessed with the Beck Anxiety Inventory (BAI) [41], which also does not include any pain items. The BAI is a 27-item questionnaire ranging from 0 to 63 also with high internal consistency (Cronbach’s α  = 0.92). A score of 0–9 refers to normal severity, whereas a score of 10–18 refers to mild severity, a score of 18–29 refers to moderate severity, and a score higher than 29 refers to severe anxiety symptoms [42].

#### Covariates

Covariates were selected a priori based on previous research on the association of depression and anxiety with pain. Socio-demographic factors included gender, age, level of education, and partner status. Furthermore, the presence of chronic diseases was taken into account as a covariate. Based on self-report during the initial interview, the presence of a chronic disease was assessed. These chronic diseases were then categorised, by a physician, into cardio respiratory disease (coronary heart disease, angina pectoris, heart failure, chronic nonspecific lung disease, stroke, hypertension), gastro-intestinal disease (diabetes, (gastro-intestinal) ulcer, ulcerative colitis or Crohn’s disease, liver cirrhosis, hepatitis) and musculoskeletal disease (arthritis, osteoarthritis, rheumatism). Because medication can have an analgesic influence on pain, the use of antidepressants and other psychotropic drugs were also selected as covariates. Also, the number of depressive episodes was taken into account as a covariate.

#### Statistical Analyses

All statistical analyses were performed in SPSS 19 for Windows. Descriptive analyses were used to assess baseline characteristics across the total sample. To assess the associations of type of disorder with the CPG, we used multinomial logistic regression analyses. For the pain outcome variable (the CPG) we used the group with no pain (CPG0) as a reference category. For type of disorder, the healthy control group without depression or anxiety was selected as a reference category. In this analysis we controlled for all the covariates.

We used adjusted multinomial analyses to assess the association of severity of depressive and anxiety symptoms with the outcome variable CPG, with the lowest severity category as the reference category.

Furthermore, we used four separate logistic regression analyses to examine the association of depression and/or anxiety with the outcome variable of location of pain (1. no pain, 2. musculoskeletal pain (controlling for the presence of musculoskeletal disease), 3. gastro-intestinal pain (controlling for the presence of gastro-intestinal disease), and 4. cardio respiratory pain (controlling for the presence of cardio respiratory disease)). Here also, having no depressive or anxiety disorder was used as a reference category.

## Results

Table 1 presents the baseline characteristics of the total population. Of the total sample, 652 participants reported no psychopathology, and slightly less participants (628) reported a remitted depressive and/or anxiety disorder (with a mean of 1.73 depressive episodes). The least participants had a current depressive disorder (with a mean of 10.29 depressive episodes), followed by a current anxiety disorder. Most participants reported a current comorbid depressive and anxiety disorder, with a mean of almost 10 depressive episodes. 26.4% of the total sample used antidepressant medication. Of the 2981 participants, 170 (5.7%) reported no pain symptoms. Most participants (92.4%) reported having musculoskeletal pain, followed by gastro-intestinal pain (1432 participants), and cardio respiratory pain (764 participants). Table 2 shows the pain characteristics, separated in no psychopathology, remitted disorder, current depressive disorder, current anxiety disorder, and current depressive and anxiety disorder. Of the total sample and of each of the abovementioned groups, most participants had low intensity and low pain-related disability (CPG1), and pain of musculoskeletal origin. Especially when a depressive disorder is comorbid with an anxiety disorder, more participants report highly disabling and severely limiting pain (CPG4).

**Table 1:** Baseline characteristics of total NESDA sample (N = 2981).

		N (%)	Mean (SD)
**Demographics**			
Female gender		1979 (66.4)	
Age in years			41.9 (13.1)
Level of education	Basic	199 (6.7)	
	Intermediate	1736 (58.2)	
	High	1046 (35.1)	
Partner or married		2066 (69.3)	
**Psychopathology characteristics**			
No psychopathology		652 (21.9)	
Remitted disorder		628 (21.0)	
	# of depressive episodes		1.73 (5.9)
Current depressive disorder		396 (13.3)	
	# of depressive episodes		10.29 (71.3)
Current anxiety disorder		543 (18.2)	
Current depressive and anxiety disorder		762 (25.6)	
	# of depressive episodes		9.88 (72.6)
Severity of depression (QIDS)*	None	1121 (37.6)	
	Mild	820 (27.5)	
	Moderate	627 (21.0)	
	(Very) Severe	374 (12.5)	
Severity of anxiety (BAI)**	Normal	1477 (49.5)	
	Mild	758 (25.4)	
	Moderate	493 (16.5)	
	Severe	218 (7.3)	
**Other characteristics**			
Musculoskeletal chronic disease		648 (21.7)	
Gastro-intestinal chronic disease		335 (11.2)	
Cardio respiratory chronic disease		775 (26.0)	
Antidepressant use		785 (26.4)	
Use of other psychotropic drugs		700 (23.5)	

*39 missing.

**35 missing.

**Table 2:** Baseline pain characteristics divided by psychopathology.

		Total	No psychopathology	Remitteddisorder	Currentdepressivedisorder	Currentanxietydisorder	Currentdepressiveandanxietydisorder
		N = 2981	N = 652	N = 627	N = 396	N = 543	N = 762
CPG*	Grade 0, N (%)	170 (5.7)	82 (12.6)	33 (5.3)	19 (4.8)	23 (4.2)	13 (1.7)
	Grade 1, N (%)	1635 (54.8)	441 (67.6)	379 (60.4)	194 (49.0)	315 (58.0)	306 (40.2)
	Grade 2, N (%)	605 (20.3)	81 (12.4)	134 (21.4)	86 (21.7)	118 (21.7)	186 (24.4)
	Grade 3, N (%)	311 (10.4)	29 (4.4)	48 (7.7)	60 (15.2)	52 (9.6)	122 (16.0)
	Grade 4, N (%)	259 (8.7)	19 (2.9)	33 (5.3)	37 (9.3)	35 (6.4)	135 (17.7)
Pain location	No pain, N (%)	170 (5.7)	82 (12.6)	33 (5.3)	19 (4.8)	23 (4.2)	13 (1.7)
	Musculoskeletal, N (%)	2753 (92.4)	552 (84.7)	586 (93.3)	374 (94.4)	507 (93.4)	734 (96.3)
	Gastro-intestinal, N (%)	1432 (48.0)	207 (31.7)	250 (39.8)	216 (54.5)	278 (51.2)	481 (63.1)
	Cardio respiratory, N (%)	764 (25.6)	77 (11.8)	117 (18.6)	101 (25.5)	156 (28.7)	313 (41.1)

*1 missing.

### Association of depression and/or anxiety with the CPG

Table 3 shows results from the adjusted multinomial logistic regression analysis assessing the association of depression and/or anxiety with the CPG. The results show a significant association and the odds ratios (ORs) rise per CPG level. For all CPG levels, the ORs were significantly elevated compared to the reference group. Having a co-morbid depressive and anxiety disorder, as compared to having no disorder, showed the strongest association (OR = 30.26; 95% CI = 12.68–72.23). Also, when compared to having no depression or anxiety, there is still a high odds of having disabling pain symptoms when the depressive or anxiety disorder is remitted (CPG1: OR = 1.87; 95% CI = 1.17–2.97; CPG4: OR = 3.53; 95% CI = 1.67–7.43). However, the confidence intervals do overlap. Therefore, four sensitivity analyses were performed, each with an other psychopathology group (remitted disorder, current depressive disorder, current anxiety disorder, comorbid depression and anxiety) as a reference category, in order to examine the possible differences in associations between pain and various depressive and anxiety disorder categories (Tables 4–7). With a current depressive disorder or anxiety disorder as reference group, the results show no significant differences between these disorders on the CPG. A current anxiety disorder and a current depressive disorder also show no significant difference with a remitted disorder. Only a co-morbid depressive and anxiety disorder had a significantly higher association with the CPG compared to a remitted, current depressive, and current anxiety disorder. These findings were similar to those in the analysis with the reference group of healthy controls. The unadjusted results did not differ from the adjusted results.

**Table 3:** Associations of presence of current depressive disorder, current anxiety disorder, current co-morbid depression and anxiety and remitted depression or anxiety with the Chronic Pain Grade, with no depression and/or anxiety disorder as a reference category.

	CPG1^a^^,^ b	CPG2^a^^,^ b	CPG3^a^^,^ b	CPG4^a^^,^ b
	*OR (95% CI)*	*OR (95% CI)*	*OR (95% CI)*	*OR (95% CI)*
No psychopathology	reference	reference	reference	reference
Remitted depression/anxiety	1.87 (1.17–2.97)*	3.45 (2.02–5.87)**	3.49 (1.81–6.73)**	3.53 (1.67–7.43)*
Depression current	1.35 (0.72–2.55)	3.53 (1.76–7.08)**	7.14 (3.28–15.54)**	6.67 (2.81–15.88)**
Anxiety current	2.10 (1.25–3.54)*	4.06 (2.26–7.30)**	5.15 (2.57–10.31)**	4.84 (2.22–10.57)**
Co-morbid depression and anxiety current	3.13 (1.56–6.25)*	10.18 (4.87–21.26)**	19.72 (8.77–44.35)**	30.26 (12.68–72.23)**

*p<.05;

**p<.001.

a reference category is no pain.

b adjusted for age, gender, level of education, partner status, antidepressant use, use of other psychotropic drugs, chronic diseases, and number of depressive episodes.

**Table 4:** Associations of presence of current depressive disorder, current anxiety disorder, current co-morbid depression and anxiety and remitted depression or anxiety with the Chronic Pain Grade, with remitted disorder as a reference category.

	CPG1^a^^,^ b	CPG2^a^^,^ b	CPG3^a^^,^ b	CPG4^a^^,^ b
	*OR (95% CI)*	*OR (95% CI)*	*OR (95% CI)*	*OR (95% CI)*
Remitted depression/anxiety	reference	reference	reference	reference
No psychopathology	.54 (.34–.85)*	.29 (.17–.50)**	.29 (.15–.55)**	.28 (.14–.60)*
Depression current	.72 (.39–1.36)	1.03 (.52–2.01)	2.04 (.98–4.25)	1.89 (.85–4.20)
Anxiety current	1.13 (.64–2.00)	1.18 (.64–2.18)	1.47 (.74–2.94)	1.37 (.65–2.91)
Co-morbid depression and anxiety current	1.68 (.84–3.36)	2.95 (1.43–6.09)*	5.65 (2.61–12.22)**	8.58 (3.84–19.21)**

*p<.05;

**p<.001.

a reference category is no pain.

b adjusted for age, gender, level of education, partner status, antidepressant use, use of other psychotropic drugs, chronic diseases, and number of depressive episodes.

**Table 5:** Associations of presence of current depressive disorder, current anxiety disorder, current co-morbid depression and anxiety and remitted depression or anxiety with the Chronic Pain Grade, with current depressive disorder as a reference category.

	CPG1^a^^,^ b	CPG2^a^^,^ b	CPG3^a^^,^ b	CPG4^a^^,^ b
	*OR (95% CI)*	*OR (95% CI)*	*OR (95% CI)*	*OR (95% CI)*
Depression current	reference	reference	reference	reference
No psychopathology	.74 (.39–1.40)	.28 (.14–.57)**	.14 (.0–31)**	.15 (.06–.36)**
Remitted depression/anxiety	1.38 (.74–2.59)	.98 (.50–1.91)	.49 (.24–1.02)	.53 (.24–1.17)
Anxiety current	1.56 (.79–3.05)	1.15 (.56–2.35)	.72 (.34–1.54)	.73 (.32–1.65)
Comorbid depression and anxiety current	2.31 (1.10–4.85)*	2.88 (1.34–6.21)*	2.76 (1.25–6.09)*	4.53 (2.00–10.30)**

*p<.05;

**p<.001.

a reference category is no pain.

b adjusted for age, gender, level of education, partner status, antidepressant use, use of other psychotropic drugs, chronic diseases, and number of depressive episodes.

**Table 6:** Associations of presence of current depressive disorder, current anxiety disorder, current co-morbid depression and anxiety and remitted depression or anxiety with the Chronic Pain Grade, with current anxiety disorder as a reference category.

	CPG1^a^^,^ b	CPG2^a^^,^ b	CPG3^a^^,^ b	CPG4^a^^,^ b
	*OR (95% CI)*	*OR (95% CI)*	*OR (95% CI)*	*OR (95% CI)*
Anxiety current	reference	reference	reference	reference
No psychopathology	.48 (.28–.80)*	.25 (.14–.44)**	.19 (.10–.39)**	.21 (.10–.45)**
Remitted depression/anxiety	.89 (.450–1.57)	.85 (.46–1.57)	.68 (.34–1.35)	.73 (.34–1.54)
Depression current	.64 (.33–1.26)	.87 (.43–1.78)	1.39 (.65–2.97)	1.38 (.61–3.13)
Comorbid depression and anxiety current	1.49 (.72–3.06)	2.51 (1.18–5.30)*	3.83 (1.74–8.42)*	6.25 (2.76–14.15)**

*p<.05;

**p<.001.

a reference category is no pain.

b adjusted for age, gender, level of education, partner status, antidepressant use, use of other psychotropic drugs, chronic diseases, and number of depressive episodes.

**Table 7:** Associations of presence of current depressive disorder, current anxiety disorder, current co-morbid depression and anxiety and remitted depression or anxiety with the Chronic Pain Grade, with comorbid depressive- and anxiety disorder as a reference category.

	CPG1^a^^,^ b	CPG2^a^^,^ b	CPG3^a^^,^ b	CPG4^a^^,^ b
	*OR (95% CI)*	*OR (95% CI)*	*OR (95% CI)*	*OR (95% CI)*
Comorbid depression and anxiety current	reference	reference	reference	reference
No psychopathology	.32 (.16–.64)*	.10 (.05–.21)**	.05 (.02–.11)**	.03 (.01–.08)**
Remitted depression/anxiety	.60 (.30–1.20)	.34 (.16–.70)*	.18 (.08–.38)**	.12 (.05–.26)**
Depression current	.43 (.21–.91)*	.35 (.16–.75)*	.36 (.16-.80)*	.22 (.10–.50)**
Anxiety current	.67 (.33–1.39)	.40 (.19–.85)*	.26 (.12–.57)*	.16 (.07–.36)**

*p<.05;

**p<.001.

a reference category is no pain.

b adjusted for age, gender, level of education, partner status, antidepressant use, use of other psychotropic drugs, chronic diseases, and number of depressive episodes.

Because the reference group for pain (CPG0) was small (N = 170), another sensitivity analysis was conducted where CPG0 and CPG1 were combined to form the reference category (Table 8). This analysis showed that the association between type of disorder and CPG mostly remains: as can be seen in table 8, all associations became less strong after combining CPG0 and CPG1 as a reference category, but remained significant.

**Table 8:** (Results from sensitivity analysis, with CPG0 and CPG1 combined) Associations of presence of current depressive disorder, current anxiety disorder, current co-morbid depression and anxiety and remitted depression or anxiety with the Chronic Pain Grade, with no depression and/or anxiety disorder as a reference category.

	CPG2^a^^,^ b	CPG3^a^^,^ b	CPG4^a^^,^ b
	*OR (95% CI)*	*OR (95% CI)*	*OR (95% CI)*
No psychopathology	reference	reference	reference
Remitted depression/anxiety	1.99 (1.45–2.73)**	2.01 (1.22–3.30)*	2.03 (1.10–3.73)*
Depression current	2.78 (1.93–4.01)**	5.61 (3.39–9.29)**	5.23 (2.77–9.85)**
Anxiety current	2.09 (1.50–2.92)**	2.65 (1.61–4.35)**	2.48 (1.34–4.59)*
Comorbid depression and anxiety current	3.63 (2.61–5.04)**	7.01 (4.38–11.23)**	10.72 (6.07–18.96)**

*p<.05;

**p<.001.

a reference category is no pain.

b adjusted for age, gender, level of education, partner status, antidepressant use, use of other psychotropic drugs, chronic diseases, and number of depressive episodes.

### Association of severity of depressive and anxiety symptoms with the CPG

Table 9 shows the association of the severity of depressive symptoms (as measured with the QIDS) and anxiety symptoms (as measured with the BAI) with the CPG. Similar to the main finding, as the severity of the depressive symptoms increases, the odds of having highly disabling and severely limiting pain increases as well. The same accounts for the association between severity of anxiety symptoms and the CPG. The unadjusted results did not differ from the adjusted results.

**Table 9:** Association of severity of depressive symptoms and anxiety symptoms with the Chronic Pain Grade.

		CPG1^a^^,^ b	CPG2^a^^,^ b	CPG3^a^^,^ b	CPG4^a^^,^ b
		*OR (95% CI)*	*OR (95% CI)*	*OR (95% CI)*	*OR (95% CI)*
QIDS^b^	None	reference	reference	reference	reference
	Mild	2.59 (1.55–4.34)**	3.72 (2.13–6.49)**	3.99 (2.13–7.49)**	4.05 (1.99–8.23)**
	Moderate	1.10 (0.59–2.02)	1.56 (.91–3.40)	3.32 (1.62–6.80)*	2.91 (1.31–6.48)*
	(Very) Severe	1.11 (.43–2.87)	2.18 (.81–5.87)	4.66 (1.65–13.16)*	7.93 (2.70–23.35)**
BAI^c^	Normal	reference	reference	reference	reference
	Mild	1.76 (.59–12.51)	2.41 (1.36–4.29)*	2.66 (1.42–4.97)*	3.07 (1.54–6.12)*
	Moderate	2.23 (.99–5.03)	4.22 (1.82–9.79)*	3.89 (1.60–9.45)*	6.69 (2.66–16.84)**
	Severe	2.73 (1.02–3.02)*	6.70 (1.44–31.20)*	8.29 (1.73–39.59)*	13.32 (2.73–64.97)*

*p<.05;

**p<.001.

a reference category is no pain.

b adjusted for age, gender, level of education, partner status, chronic diseases, antidepressant use, use of other psychotropic drugs, number of depressive episodes, and severity of anxiety symptoms.

c adjusted for age, gender, level of education, partner status, chronic diseases, antidepressant use, use of other psychotropic drugs, number of depressive episodes, and severity of depressive symptoms.

Because the reference group for pain (CPG0) was small (N = 170), a sensitivity analysis was conducted where CPG0 and CPG1 were combined to form the reference category (table 10). This analysis showed that the association found between severity of depressive and anxiety symptoms and CPG mostly remains, with more severe depressive or anxiety symptoms being more strongly associated with more disabling and limiting pain, when CPG0 and CPG1 were combined to have a larger reference group. Here also, all associations were less strong, with some associations (especially the association of severe anxiety symptoms with the CPG2 and CPG4) remaining statistically significant, while other associations (e.g. the association of mild and moderate anxiety symptoms with CPG3) becoming non-significant.

**Table 10:** (Results of sensitivity analysis, with CPG0 and CPG1 combined) Association of severity of depressive symptoms and anxiety symptoms with the Chronic Pain Grade.

		CPG2^a^^,^ b	CPG3^a^^,^ b	CPG4^a^^,^ b
		*OR (95% CI)*	*OR (95% CI)*	*OR (95% CI)*
QIDS^b^	None	reference	reference	reference
	Mild	1.55 (1.18–2.02)*	1.65 (1.11–2.46)*	1.67 (1.00–2.80)*
	Moderate	1.67 (1.21–2.33)*	3.14 (2.04–4.83)**	2.75 (1.58–4.78)**
	(Very) Severe	2.05 (1.36–3.09)*	4.37 (2.61–7.33)**	7.42 (4.08–13.649)**
BAI^c^	Normal	reference	reference	reference
	Mild	1.42 (1.09–1.85)*	1.57 (1.09–2.24)*	1.81 (1.14–2.87)*
	Moderate	1.99 (1.44–2.75)**	1.83 (1.19–2.81)*	3.15 (1.91–5.19)**
	Severe	2.59 (1.63–4.13)**	3.12 (1.86–5.53)**	5.16 (2.83–9.41)**

*p<.05;

**p<.001.

a reference category is no pain.

b adjusted for age, gender, level of education, partner status, chronic diseases, antidepressant use, use of other psychotropic drugs, number of depressive episodes, and severity of anxiety symptoms.

c adjusted for age, gender, level of education, partner status, chronic diseases, antidepressant use, use of other psychotropic drugs, number of depressive episodes, and severity of depressive symptoms.

### Association of depression and/or anxiety with musculoskeletal, gastro-intestinal, and cardio respiratory pain symptoms

Table 11 shows the multinomial logistic regression analyses assessing the association of depression and/or anxiety with three clustered pain locations. For those with pain, the highest ORs are seen in co-morbid depression and anxiety. The ORs for musculoskeletal pain range from 2.28 (95% CI = 1.53–3.42) for a depressive or anxiety disorder in remission to 3.88 (95% CI = 2.33–6.47) for a co-morbid depressive and anxiety disorder. For gastro-intestinal pain, the ORs range from 1.40 (95% CI = 1.10–1.78) for a depressive or anxiety disorder in remission to 3.31 (95% CI = 2.57–4.27) for a co-morbid depressive and anxiety disorder. Cardio respiratory pain shows a range in ORs from 1.74 (95% CI = 1.27–2.40) for a depressive or anxiety disorder in remission to 5.15 (95% CI = 3.80–6.98) for co-morbid depressive and anxiety disorder. The unadjusted results did not differ from the adjusted results.

**Table 11:** Associations of remitted psychopathology, current depressive disorder, current anxiety disorder, and current co-morbid depression and anxiety with pain location, with no depression and/or anxiety as reference category.

	No pain^a^	Musculoskeletal^a^	Gastro-intestinal^a^	Cardio respiratory^a^
	*OR (95% CI)*	*OR (95% CI)*	*OR (95% CI)*	*OR (95% CI)*
No psychopathology	reference	reference	reference	reference
Remitted depression/anxiety	.47 (.30–.75)*	2.28 (1.53–3.42)**	1.40 (1.10–1.78)*	1.74 (1.27–2.40)*
Depression current	.52 (.28–.97)*	2.64 (1.53–4.56)**	2.43 (1.84–3.22)**	2.63 (1.86–3.70)**
Anxiety current	.39 (.24–.66)**	2.29 (1.49–3.51)**	2.08 (1.61–2.68)**	2.96 (2.16–4.04)**
Co-morbid depression and anxiety current	.19 (.10–.38)**	3.88 (2.33–6.47)**	3.31 (2.57–4.27)**	5.15 (3.80–6.98)**

*p<.05;

**p<.001.

a = adjusted for age, gender, level of education, partner status, antidepressant use, use of other psychotropic drugs, number of depressive episodes, and chronic disease.

## Discussion

The high proportion of participants with anxiety and depressive disorders in this study reflects the sampling strategy for including sufficient numbers of respondents to examine individuals at different stages of development and severity of depression and anxiety. This study demonstrates considerable associations between presence of depressive and anxiety disorders (current and remitted) and symptom severity with different pain dimensions, namely pain-related disability, pain intensity, and the location of pain symptoms (musculoskeletal, gastro-intestinal, and cardio respiratory).

### Presence and severity of depressive or anxiety disorder

Our results show that having a mood or anxiety disorder increases the odds of highly disabling and severely limiting pain. Also, the severity of the depressive and anxiety symptoms are significantly associated with pain-related disability and limiting pain, with more severe symptoms having higher odds for highly disabling and severely limiting pain.

Depressive and anxiety disorders may add to pain as they increase the likelihood of social isolation, increased attention towards threat and avoidance of physical exertion [11], [12], [16], [17]. Depression and anxiety disorders also share the same pathophysiological pathways as pain [43]–[45]. They facilitate the central modulation of the pain response, in the periaqueductal gray, amygdala, and hypothalamus [1], [13], [14], and when deficits occur in these areas, modulation of signals from the body are disturbed, leading to a more severe experience of pain. Although these brain areas all play a role in depression, anxiety, and pain, not every individual responds the same to pain stimuli [46]–[48]. Some individuals are more sensitive to pain than others. The use of EEG may help in identifying neuronal markers for sensitivity to pain [46], and whether there are differences between depressive and anxious individuals. Furthermore, depression and anxiety induce stress and increases the production of pro-inflammatory cytokines [49]–[51], which may increase pain [52], [53]. The finding showing higher pain-related disability in co-morbid depression and anxiety in our study is similar to findings from the STAR*D studies [54]–[56].

### Depressive or anxiety disorder in remission

Regarding the association of a remitted disorder and pain, we expected persons with remitted depression or anxiety to have similar pain symptoms as controls without depression or anxiety. However, this is not the case. Persons with a remitted depressive or anxiety disorder still showed high odds on the pain outcomes when compared to those that have no such disorder. This is remarkable, because this means that having a remitted disorder is not the same as having no disorder with regards to pain. This raises several questions.

First, pain symptoms may be residual symptoms in depressive and anxiety disorder. This finding may suggest that pain in patients with remitted depressive or anxiety disorder might be indicative of a risk for recurrence, as disability in life (e.g. disability to fulfil his or her role at home) is an important risk factor for the recurrence of anxiety disorders [57], and our results show that those with a remitted diagnosis still have high odds of having pain symptoms and pain-related disability.

Another explanation for the high odds of pain symptoms in remitted depression or anxiety might be that treatment, if it was provided, for the depressive or anxiety disorder is not effective for pain symptoms, or treatment should be different when pain is co-morbid with the depressive or anxiety disorder. For example, it has been suggested that patients with depression and pain are a distinctively other group of patients than those with depression without pain, with the former having a focus on health and the latter having a focus on a negative view of the self [58], [59]. If that is the case, the treatment should be different for depressive disorder with pain and depressive disorder without pain. The same could possibly apply to patients with an anxiety disorder with or without pain. This finding warrants further research, to examine if having a depressive or anxiety disorder in remission may be a possible risk factor for subsequent long lasting chronic pain.

### Pain location

Of the total sample, 92.4% reported pain symptoms of musculoskeletal nature, followed by 48% reporting gastro-intestinal pain and 25.6% of cardio respiratory pain. Because musculoskeletal pain has by far the highest prevalence in this study, we expected that this pain location would have the highest association with depression and anxiety. However, this expectation was not confirmed. Our findings show that those with a current depressive disorder or a remitted depressive or anxiety disorder have high odds for having musculoskeletal pain, but the odds for having pain of cardio respiratory origin are also large when compared to having no depressive or anxiety disorder. Furthermore, persons having a current anxiety or co-morbid depressive and anxiety disorder show high odds for having cardio respiratory pain. These findings remain after adjustment for chronic cardio respiratory illness.

In a review by Celano & Huffman [60], depressive disorder was associated with cardiac disease, such as coronary artery disease. This also applies to anxiety which appears to be a risk factor for coronary heart disease and cardiac mortality [61]. The finding that anxiety shows such high odds for cardio respiratory pain symptoms, even when we controlled for cardio respiratory disease, could be due to the fact that anxious patients with chest pain are more sensitive to bodily sensations [62]; in that case, the experience of pain would be centrally modulated in such a way as to elevate the odds for experiencing pain. However, another explanation might be possible as well; stress induced anxiety has an elevating effect on cytokines [49], and elevated cytokines lead to pain symptoms. This might explain our findings.

In the STAR*D study, depressed patients (with less sleep quality and sympathetic arousal) showed an association with higher cardiac risk, which may be similar to our findings [63] whereby having a depressive disorder had higher odds for having cardio respiratory pain when compared to having no depressive or anxiety disorder. Even when we controlled for having a chronic disease of cardio respiratory origin, the association remained strong and significant. Previous research showed that having a mental illness is a possible risk factor for cardiovascular disease [64], [65]. Our finding that a depression/anxiety, whether or not in remission, shows a strong association with cardiac pain is worth exploring in further longitudinal research to explore a possible causal relation of depression and/or anxiety (current or in remission) with cardiac disease, of which cardio respiratory pain might be an early indicator.

### Strengths of the study

A strength of this study is the large sample size. Also, because patients with a depressive or anxiety disorder often report pain in multiple locations, the categorisation of pain locations makes these results clinically relevant and widely applicable. Moreover, this study not only examines the association of pain with depressive or anxiety disorder, but also with co-morbid depression and anxiety, as well as with remitted disorders. Additionally, in the analyses of the pain symptoms, the no pain group was the reference category which is new compared to earlier studies that compared low pain with high pain – which is subject to interpretation bias as pain is a subjective experience. In our study, we had a healthy control group with no pain in which better contrasts could be made with those who had pain.

### Limitations

This study also has some limitations. First, it is not possible to make inferences about causality because this was a cross-sectional observational study. Second, some of the subgroups of our sample were smaller than the other groups, such as those with no pain symptoms (CPG0, N = 170). However, a sensitivity analysis revealed that the association between severity of depressive and anxiety symptoms and CPG mostly remains, with more severe depressive or anxiety symptoms being more strongly associated with more disabling and limiting pain when CPG0 and CPG1 were combined to have a larger reference group. Therefore, these associations can be considered valid and of clinical relevance as they indicate a clear difference in association with depression or anxiety between patients with and without pain. Another possible limitation is the self-reporting of physical illness, which might lead to overreporting or underreporting of chronic physical illness; however, a study by Kriegsman et al. [66] shows that patients report their physical illness fairly accurately when compared to the reports of their general practitioner, even when taking depressive symptomatology into account. Furthermore, no information was available whether a physical illness was organic or functional, which may have its effect on pain. For example, individuals with an organic disease describe their pain as consistent, whereas those without an organic disease describe their pain as variable and diffuse [67]. Also, PTSD has been linked to increased risk for pain [68], [69]. Therefore, future research that examines the association of anxiety disorders with pain should also include PTSD.

## Conclusion

This study shows that depressive and anxiety disorders have a similar and very strong association with the CPG (which includes pain-related disability and pain intensity) and musculoskeletal pain, cardio respiratory pain, and gastro-intestinal pain compared to a control group without depressive or anxiety disorder. Depression and anxiety share the same pathophysiological pathways as pain and can have a reciprocal effect on each other, which could explain these associations. Moreover, even a remitted disorder has a strong association with pain. This might mean that patients with depression or anxiety and pain are a different group and need different treatment than patients that do not have pain accompanying their depression or anxiety. Depression and anxiety also have a strong association with cardio respiratory pain, and this association remained after correction for cardiovascular or respiratory illness. This strong association between depression/anxiety (current or in remission) with cardio respiratory pain is an interesting finding, which warrants further longitudinal research to examine a possible causal relation of cardiac pain and a mental disorder (current or in remission) with cardiac disease.
