# Correction: The Association of Depression and Anxiety with Pain: A Study from NESDA

**DOI:** 10.1371/journal.pone.0115077

**Published:** 2014-12-01

**Authors:** The PLOS ONE Staff

In the Author Contributions section, Christina M. van der Feltz-Cornelis (CMFC) should be listed as one of the persons who analyzed the data.
